# Modulating Electrical Properties of Ti64/B_4_C Composite Materials via Laser Direct Manufacturing with Varying B_4_C Contents

**DOI:** 10.3390/ma17174184

**Published:** 2024-08-23

**Authors:** Wenshu Zhang, Hui Chang, Ning Dang, Lian Zhou

**Affiliations:** 1 College of Materials Science and Engineering, Nanjing Tech University, Nanjing 211816, China; wenshu@njtech.edu.cn; 2 Research & Development Institute of Northwestern Polytechnical University in Shenzhen, Shenzhen 518063, China; dangning87@hotmail.com

**Keywords:** Ti64/B_4_C composite materials, laser direct manufacturing, electrical properties, electromagnetic shielding, dielectric polarization

## Abstract

The modulation of electrical properties in composite materials is critical for applications requiring tailored electrical functionality, such as electromagnetic shielding and absorption. This study focuses on Ti64/B_4_C composites, a material combination promising enhanced electromagnetic properties. Laser direct manufacturing (LDM) was utilized to fabricate coaxial samples of Ti64 blended with TiB and TiC in various mass ratios, with sample thicknesses ranging from 0.5 mm to 3.5 mm. The electrical characterization involved assessing the dielectric and magnetic permeability, as well as impedance and reflectance, across a frequency spectrum of 2 to 18 GHz. The result reveals that TiC, when incorporated into Ti64, exhibits strong dielectric polarization and achieves a reflectivity as low as −40 dB between 7 and 14 GHz. Conversely, TiB demonstrates effective electromagnetic absorption, with reflectivity values below −10 dB in the frequency band of 8.5 to 11.5 GHz. The study also notes that a lower B_4_C content enhances electronic polarization and increases the dielectric coefficient, while higher contents favor ionic polarization. This shift can lead to a timing mismatch in the establishment of electron and ion polarization, resulting in a decreased dielectric coefficient. In addition, adjusting the B_4_C content in Ti64/B_4_C composites effectively modulates their electrical properties, suggesting a strategic approach to designing materials for specific electromagnetic functions.

## 1. Introduction

Advanced composite materials with tailored electrical properties are increasingly vital in technological applications such as electromagnetic shielding and absorption. Titanium and its alloys, such as Ti64, are renowned for their exceptional strength-to-weight ratios, corrosion resistance, and biocompatibility, making them indispensable in aerospace, biomedical, and marine applications [1,2,3]. The integration of B_4_C into Ti64 alloys via innovative manufacturing techniques promises advancements in material properties that can be tuned for specific functional requirements [4,5].

Recent advancements in additive manufacturing (AM) techniques, such as laser powder bed fusion (LPBF) and laser direct manufacturing (LDM, same as LDED), and microstructural control has ushered in novel avenues for tailored customization of titanium alloy properties [6,7,8].

To enhance the LDM preparation of titanium’s performance, many efforts have been explored to enhance Ti64 alloys and improve the electrical conductivity and shielding performance, such as enhancing its strength [9,10], hardness [11], elastic modulus [12,13], heat resistance [14], and corrosion resistance [10]. This is attributed to B_4_C’s ability to bolster Ti64 through particle strengthening and forming TiB and TiC reinforcing phases in situ via alloy reactions [15,16], thereby broadening its application spectrum [16,17]. During the additive manufacturing process of Ti64, in-situ-generated TiB [18] and TiC [5,17,19] exhibit superior wave-absorbing properties. However, there have been no reported studies investigating whether using in-situ-generated TiB and TiC at same time in manufacturing Ti64 can enhance the wave-absorbing capabilities of this material.

Despite considerable progress, there remains a gap in the comprehensive understanding of the interactive effects of B_4_C particle size [20], distribution, and concentration on the microstructure and properties of titanium alloys produced via additive manufacturing [21,22,23]. Additionally, the impact of these modifications on the functional properties like electromagnetic interference (EMI) shielding and high-temperature behavior has not been exhaustively explored in the context of AM processes.

This paper aims to build on existing research by providing a detailed study of the effects of various B_4_C addition strategies on the microstructure, mechanical, and functional properties of Ti64 alloys fabricated using LDM techniques, and examines how different B_4_C particle sizes and concentrations influence the alloy’s performance across a range of applications, from aerospace components to biomedical implants. Additionally, this study will delve into the thermal and electromagnetic properties of the composites, providing insights into their potential use in high-temperature and EMI-sensitive environments. Through a combination of experimental analysis and predictive modeling, this research seeks to establish a set of guidelines for optimizing B_4_C-reinforced titanium alloys for specific applications, thereby contributing to the advancement of material engineering and additive manufacturing technology.

## 2. Materials and Methods

### 2.1. Materials

The sample raw materials are selected with the Ti64 powder (Al:5.78, V:4.06, Fe:0.23, O:0.068) wt.% and a particle size of 50~150 µm; B_4_C, TiB, and TiC are all selected with a purity of 99.9% and a particle size of 20~50 µm. In order to verify the influence of the ratio of Ti64 to TiB, TiC, and B_4_C on electromagnetic parameters, Ti64: B_4_C, Ti64: TiB, and Ti64: TiC were uniformly mixed according to the ratios in Table 1, Table 2 and Table 3, and then mixed in a ratio of 7:3 between the mass of the mixed powder and the mass of paraffin to prepare a 3.5 mm coaxial ring test sample.

### 2.2. Method

Although LDM technology can effectively reduce the manufacturing cost of Ti64 alloys, in terms of performance, the Ti64 alloy produced by LDM manufacturing technology is inferior to that produced by traditional forging technology in terms of strength, hardness, elastic modulus, and corrosion resistance. In order to improve the performance of Ti64 prepared by LDM, researchers introduced B into Ti64 titanium alloy_4._ The C powder particles effectively improve the strength, hardness, elastic modulus, high temperature resistance, and corrosion resistance of Ti64. The main reason for this is B_4_C itself can not only enhance particles inside Ti64, but also generate in situ TiB and TiC reinforcement complexes in Ti64 through chemical reactions in the titanium alloy, as shown in Equation (1), during the printing process. This makes it possible to use the LDM method to process Ti64 parts with complex shapes [15], further expanding the application field of Ti64.

(1) $$5Ti+B_{4}C\to 4TiB+TiC$$

Due to the presence of hexagonal phases in Ti64 alloy α- Ti and body-centered cubic phase β- Ti, the different lattice structures of the two determine their corresponding electrical conductivity. Therefore, according to Equation (2), the dielectric coefficients of the two phases are different and can change at different electric field frequencies. On the one hand, at the interface between the two phases of Ti64, electromagnetic waves can undergo multiple reflections and refractions, making it difficult for electromagnetic waves entering the interior of Ti64 to reflect or transmit smoothly in order to reduce the reflectivity of electromagnetic waves. On the other hand, different phases with different dielectric constants have different polarization mechanisms and polarizabilities within their lattice. Under the combined action of the electromagnetic field and interface, the polarizability inside the two phases can be changed or even cause new polarization forms, thereby affecting the overall electromagnetic wave reflectivity of Ti64. Through the above two analyses, it is shown that Ti64 itself can be used as an absorbing material, but there are few reports on the electromagnetic parameter measurement of Ti64 [24].

(2) $$\varepsilon =\varepsilon^{\prime }+i\frac{\sigma }{\omega }\;\varepsilon =\varepsilon^{\prime }+i\varepsilon^{\prime \prime }\mu =\mu^{\prime }+i\mu^{\prime \prime }$$

In addition, TiB and TiC generated in situ during the printing process in Ti64 also exhibit excellent absorption performance. In order to further investigate the above issues, this article adds different contents of B to Ti64 powder in a certain proportion of _4_C. Measure the electromagnetic parameters of TiB and TiC nanoparticles, calculate the impedance and reflectivity of the samples, and obtain the influence of the proportional relationship between Ti64, B_4_C, TiB, and TiC on the electromagnetic parameters. At the same time, summarize whether doping with the above-mentioned compounds with different contents can improve the absorption performance of Ti64 titanium alloy to a certain extent.

**Figure 1:**
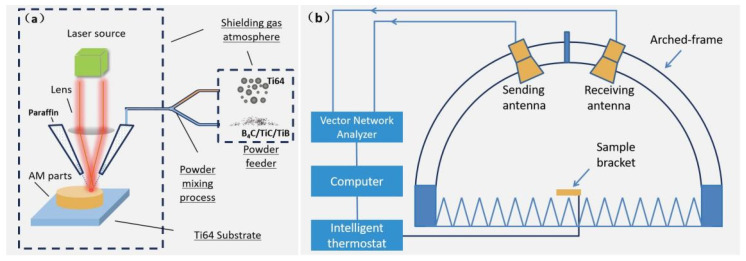
(**a**) Schematic diagram of specimen preparation processes via LDM. (**b**) Structure sketch of vector network analyzer testing processes.

As shown in Figure 1a, in the LDM system (LDM8060 made by Nanjing Zhongke Raycham Laser Technology Co., Ltd., Nanjing, China), the sintering temperature is controlled below 80 °C, so a cylindrical ring with an inner diameter of 3.0 mm and an outer diameter of 7.0 mm was prepared, and the samples can match the vector network analyzer testing. The dielectric coefficient (τ) and magnetic permeability (µ) of the sample were tested using Agilent’s E5230C vector network analyzer (2–18 GHz), and the impedance and reflectance coefficients of the above samples were calculated according to Equations (3) and (4) to analyze Ti64, B_4_C, and Ti64. The impact of TiC quality variation on electromagnetic parameters was investigated. In addition, using Equation (5), the relaxation time of the sample is most sensitive to the frequency ω of the external electric field, and at this juncture, the dielectric polarization loss reaches its maximum, and the relaxation time of each sample can be obtained τ. To determine the type of polarization in the sample, we examined ω [25]. The frequency of the external electric field [26] and the impedance matching values (Zi/Zo) and electromagnetic reflectance (RL) can be calculated at different frequencies and thicknesses based on the electromagnetic parameters obtained from testing, as shown in Equations (3) and (4). The reliability of calculating reflectance using electromagnetic parameters was verified by measuring the actual reflectance of coating samples with a size of 180 mm × 180 mm and a measured coating thickness of 2 mm.

(3) $$Z_{i}=Z_{0}\sqrt{\frac{\mu_{r}}{\varepsilon_{r}}}\tanh\left[\frac{i\left(2\pi fd\sqrt{\mu_{r}\varepsilon_{r}}\right)}{c}\right]$$

(4) $$RL\left(dB\right)=20\mathrm{lg}\left|\frac{Z_{i}-Z_{0}}{Z_{i}+Z_{0}}\right|$$

(5) ω∗τ=1

## 3. Result and Discussion

### 3.1. Electromagnetic Performance of Ti64/BC4 Mixture

**Figure 2:**
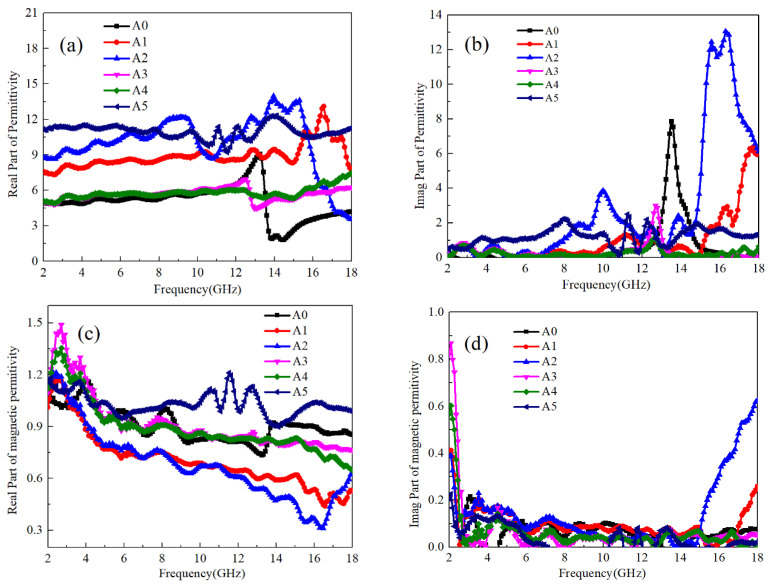
The electromagnetic parameters of Ti64/B_4_C with different ratios: (**a**) real part of dielectric coefficient, (**b**) imaginary part of dielectric coefficient, (**c**) real part of magnetic permeability, (**d**) imaginary part of magnetic permeability.

The measurement results of the real and imaginary parts of the dielectric coefficients and magnetic permeability of Ti64 and B_4_C with different mass ratios are shown in Figure 2. It can be seen that the dielectric coefficient of pure Ti64 (A0) sample is relatively low below 12 GHz, about 5. At 12 GHz~14 GHz, the real part of the dielectric coefficient first increases to about 8, and then decreases to around 2. Combined with Figure 2b, the imaginary part of the dielectric coefficient can be obtained. Ti64 has the highest imaginary part of the dielectric coefficient in this frequency band, indicating the existence of anomalous dispersion phenomenon in the sample. According to Equation (5), it can be obtained that the electron polarization establishment time of pure Ti64 sample is 7.2 × 10^−11^ s. For pure B_4_C samples, there is no significant change in the value of dielectric coefficient in the range of 2–18 GHz, which is about 11. Combined with the imaginary part value of dielectric, it can be found that there are maximum values at 8 GHz and 15 GHz, and the polarization establishment time for both is calculated to be 1.3 × 10^−10^ s and 6.6 × 10^−11^ s, respectively. Due to the NaCl-type simple cubic structure of B_4_C [27], there is a possibility of ion polarization between B and C atoms. Additionally, due to the uneven distribution of electron clouds between B and C atoms, two types of polarization can occur. As ion polarization takes slightly longer to establish than electron polarization, it corresponds to ion polarization of B_4_C at 8 GHz and electron polarization at 15 GHz.

When B_4_C and Ti64 powder are mixed, when the B_4_C: Ti64 content ratio is less than 1:30, the real part of the dielectric coefficient increases in the range of 2–16 GHz with the increase of B_4_C content. The real part of the dielectric coefficient of A1 sample increases to about 7.5, and A2 increases to 9–12. When the ratio of the two is higher than 1:30, the dielectric coefficient decreases with the increase in B_4_C content and does not decrease after the content is higher than 1:10. The real part of the dielectric coefficient of A3 sample and A4 sample are both around 5.5. From the above evidence, it can be concluded that B_4_C has two polarization modes and a higher dielectric coefficient than Ti64, which indicates that B_4_C is higher than Ti64 in both ion and electron polarizabilities. At the same time, the electron polarization establishment time of the two is relatively close. According to the values of the dielectric imaginary part of A1~A4 samples, the highest values of the dielectric imaginary part of A1~A4 samples are about 6.1, 13.2, 3.0, and 1.5, respectively. The frequency of anomalous dispersion corresponds to 17.8 GHz, 16.2 GHz, 13.0 GHz, and 12.8 GHz, respectively, and the corresponding polarization establishment time is 5.6 × 10^−11^ s, 6.2 × 10^−11^ s, 7.2 × 10^−11^ s, and 7.8 × 10^−11^ s, respectively. It can be concluded that when B_4_C attaches to Ti64, as the B_4_C content increases, the internal polarization establishment time of Ti64 gradually prolongs. This also indicates that the influence of B_4_C on Ti64 polarization mode transitions from electronic polarization to ion polarization. When the B_4_C: Ti64 content ratio is 1:30, B_4_C can induce the maximum electronic polarization intensity inside Ti64. Therefore, the dielectric coefficient of A2 sample reaches its highest point. As the B_4_C content increases, it begins to affect the polar atoms inside Ti64, causing the ion polarization phenomenon to gradually appear inside Ti64. Due to the mismatch between ion polarization frequency and electron polarization at this time, the polarization forms of the two are mutually constrained and difficult to enhance. Therefore, for A3A, the dielectric coefficient of A4 samples is generally low.

**Figure 3:**
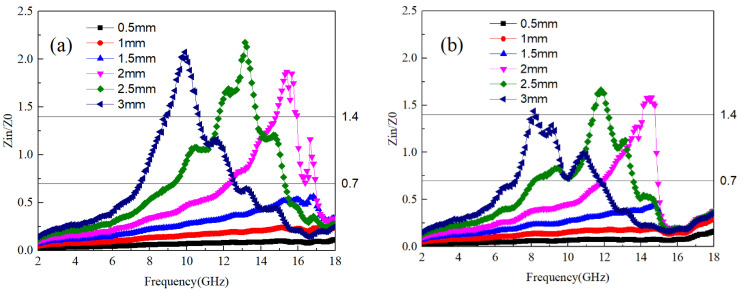

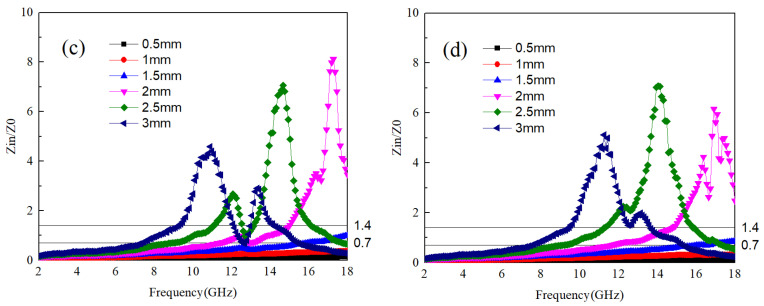
The impedance values of Ti64/B_4_C with different ratios; (**a**–**d**) correspond to the impedance values of A1~A4 samples at different thicknesses, respectively.

**Figure 4:**
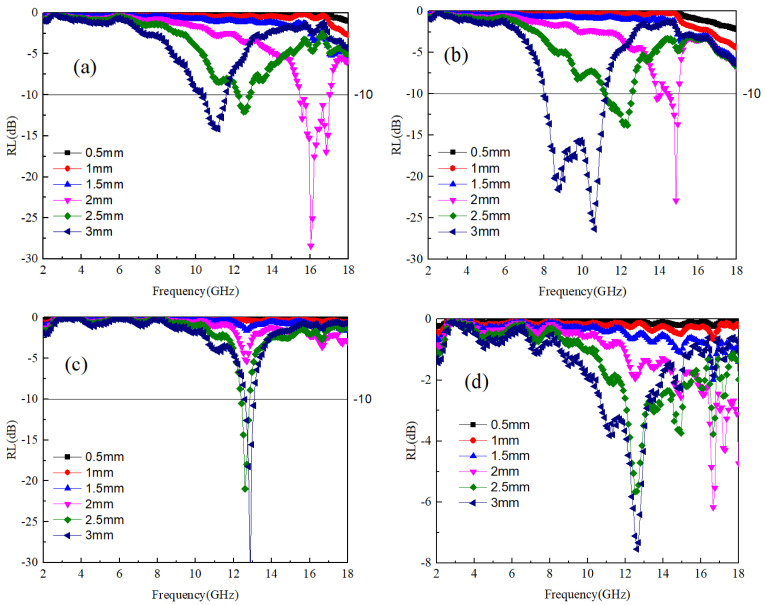
The impedance values of Ti64/B_4_C with different ratios; (**a**–**d**) correspond to the reflectivity of A1~A4 samples at different thicknesses, respectively.

As Ti64 and B_4_C are both non-magnetic materials, it can be seen from the real and imaginary parts of the magnetic permeability in Figure 2c,d that the real part of the magnetic permeability of all samples is around 1 at low frequencies, and the imaginary part is zero. The high imaginary part of magnetic permeability below 2 GHz in Figure 2d is due to the boundary effect of the instrument when processing imaginary part data, and the same phenomenon exists in Figure 3d and Figure 4d. When the frequency increases, the real part of magnetic permeability slightly decreases, while for samples A1 and A2 above 14 GHz, both the real and imaginary parts of magnetic permeability increase. Based on the observed decrease in both the real and imaginary parts of the dielectric coefficient depicted in Figure 2a,b, it is evident that the polarized electrons induced by B_4_C in Ti64 contribute significantly. This effect arises from the similar polarization times of the electrons, leading to internal resonance and electromagnetic conversion. Consequently, this enhances the sample’s sensitivity to electromagnetic wave loss [28]. Furthermore, it can be inferred that when the B_4_C and Ti64 content ratio is below 1:30, B_4_C effectively augments electronic polarization within Ti64, consequently increasing its real dielectric coefficient and expediting the polarization establishment time. However, when the content is higher than 1:30, ion polarization can affect the lattice vibration frequency of Ti64, thereby changing the electron polarization establishment time of Ti64. Therefore, abnormal dispersion phenomenon cannot be observed.

The ability of electromagnetic waves to enter the interior of a material and generate losses depends on the material’s impedance to electromagnetic waves [29]. According to Equation (2), Zn is the impedance of the sample to electromagnetic waves. The material’s impedance matching is assessed using the ratio Zn/Z0, where a value between 0.7 and 1.4 indicates favorable matching conditions. Within this range, electromagnetic waves can penetrate the material’s interior and induce electromagnetic losses. Figure 3a–d show the impedance matching values of Ti64 and B_4_C at different mass ratios. In order to obtain better application data, the impedance values of A1~A4 samples at different thicknesses are provided together. It can be seen that the frequency range with good impedance matching values for A1 and A2 samples is relatively wide. where the optimal impedance matching frequency band for A1 samples at a thickness of 3 mm is 7–9 GHz and 11–13 GHz. As the thickness of the sample decreases, the frequency band with optimal impedance matching shifts towards higher frequencies. This pattern is consistent across samples A2 to A4. Thus, it can be inferred that sample thickness exerts a certain influence on the optimal impedance matching frequency band. Specifically, thicker samples tend to have the optimal matching frequency band closer to lower frequencies. Conversely, when sample thickness is less than 2 mm, the smaller size allows electromagnetic waves to readily transmit through the material, resulting in impedance matching values consistently below 0.7.

(6) Z0=ε0μ0≈3

The impedance matching values for the A3 and A4 samples are significantly higher compared to those of the A1 and A2 samples. According to the findings from Figure 2a,b, it is evident that a low dielectric value can elevate the impedance matching value of the material to electromagnetic waves. This results in a narrowing of the frequency bandwidth with good impedance matching, which hinders the penetration of electromagnetic waves into the material’s interior. For the absorption rate of electromagnetic waves, Equation (3) is used to calculate for each sample. When the reflectivity is below −10 dB, it is considered that the sample can absorb 90% of the electromagnetic waves, which is the effective absorption frequency band. The reflectivity of samples A1~A4 is shown in Figure 4a–d. It can be seen that the A4 sample has no effective absorption frequency band in different thicknesses and the frequency band of 2–18 GHz. The lack of significant dielectric and magnetic losses in the frequency band contributes to the observed behavior of the A4 sample. Additionally, the high overall impedance matching value of the A4 sample poses challenges in generating an effective absorption frequency band. Conversely, while the A3 sample exhibits noticeable dielectric loss around 12–14 GHz, it only demonstrates the optimal impedance matching frequency band near 13 GHz based on the impedance matching value. Therefore, the A3 sample only has the best absorption frequency band near 13 GHz, and its lowest reflectivity value is −35 dB with a thickness of 3 mm. For the A1 sample, the frequency range of dielectric loss is between 10–13 GHz and 14–18 GHz, and the value of the corresponding impedance matching optimal frequency band decreases with increasing thickness. Figure 4a shows a mismatch between dielectric loss and impedance matching optimal frequency band, resulting in strong absorption at high frequencies with a reflectivity of only 2 mm and a value of −30 dB. For the A2 sample, due to the high overlap between the dielectric loss frequency band and the impedance matching optimal frequency band, this also means that within the frequency band with the maximum dielectric loss, electromagnetic waves can effectively enter the interior of the material, generating electromagnetic losses. Therefore, the electromagnetic effective loss frequency in the A2 sample is the widest, that is, under the condition of 3 mm, the absorption frequency width is 8–12 GHz, and the minimum reflectivity is −27 dB.

The observed data indicate that alterations in the mass ratio of Ti64 to B_4_C exert a notable impact on the electrical properties of the composite material. Specifically, when the Ti64:B_4_C ratio falls below 1:30, an escalation in B_4_C content corresponds to an increase in dielectric loss. Concurrently, there is a reduction in impedance within the material, resulting in an expanded bandwidth for impedance matching. Conversely, at a Ti64:B_4_C ratio of 30:1, the frequency band associated with dielectric loss aligns optimally with the impedance matching frequency band, thereby maximizing the material’s absorption capacity for electromagnetic waves. However, beyond this ratio, further increases in B_4_C content lead to a decrease in dielectric loss within the sample, accompanied by an elevation in impedance and subsequent decline in absorption capability.

### 3.2. Electromagnetic Performance of Ti64/TiB Mixture

**Figure 5:**
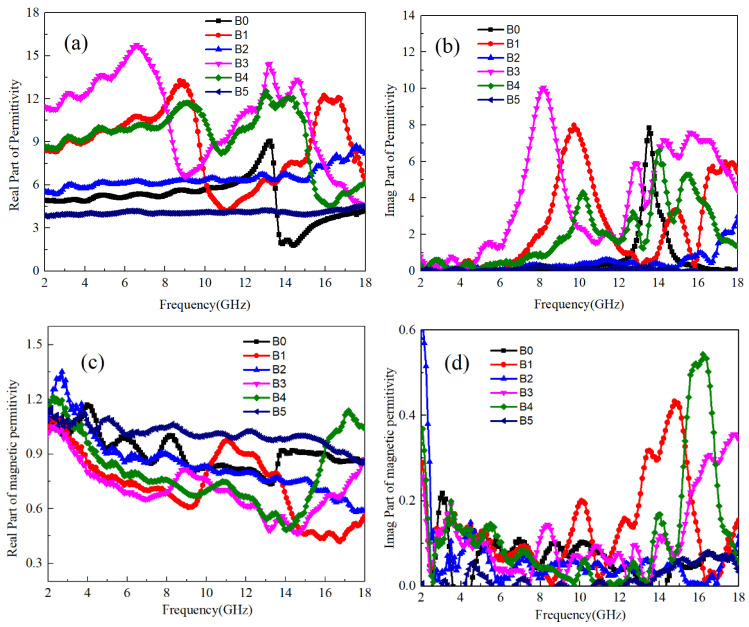
The electromagnetic parameters of Ti64/TiB with different ratios: (**a**) real part of dielectric coefficient, (**b**) imaginary part of dielectric coefficient, (**c**) real part of magnetic permeability, (**d**) imaginary part of magnetic permeability.

Figure 5a,b depict the real and imaginary components of the dielectric coefficients for Ti64 and TiB across various mass ratios. Analysis of the real part of the dielectric coefficient reveals that TiB samples exhibit negligible dielectric loss within the frequency range of 2–18 GHz. This observation suggests that the primary polarization mode of TiB is electronic polarization, characterized by an exceedingly brief establishment time for polarization. Despite TiB’s relatively weak polarization capability, its dielectric coefficient experiences a significant increase when combined with Ti64. For instance, when the TiB:Ti64 ratio is 1:100 (B1 sample), the real component of the dielectric coefficient reaches a peak value of 13 within the 2–9 GHz range, demonstrating a further increase with higher TiB content. When the TiB: Ti64 ratio is 1:30, the dielectric coefficient significantly decreases, to about 6. It is interesting that as the content of TiB is further increased, the dielectric coefficient of B3 sample gradually increases to the maximum value of 17 at 2–7 GHz. Continuing to increase the content of B_4_C, the dielectric coefficient of B4 sample begins to decrease again, and its value is closer to that of B1 sample.

Upon testing of the dielectric imaginary part of TiB and Ti64 mixture, it is observed that in the B1 sample, where the TiB content is low, the frequency corresponding to anomalous dispersion is approximately 9.8 GHz. Despite the heightened dielectric loss observed in this sample around 15.0 GHz and 16–18 GHz, a comparison with the dielectric real part reveals a simultaneous increase in both the real and imaginary components within this frequency band. This simultaneous increase indicates that the dielectric loss occurring in this frequency range does not align with anomalous dispersion but rather corresponds to resonance loss. This distinction is crucial for understanding the underlying mechanisms contributing to the electrical behavior of the composite material. Therefore, for the B1 sample, polarization only exists at 10 GHz, with a polarization establishment time of 1.0 × 10^−10^ s. When the TiB content is low, it can increase the internal electronic polarization intensity of Ti64 and prolong the polarization establishment time. For the B2 sample, there is only a weak resonance loss in the frequency range of 16–18 GHz, and there is no obvious dielectric relaxation phenomenon in the frequency range of 2–16 GHz. This means that at this ratio, TiB begins to affect the polar atomic vibration inside the Ti64 lattice. Due to the mismatch between electronic polarization frequency and polar atomic vibration, TiB itself suppresses electronic polarization inside Ti64 at this ratio, resulting in a decrease in both the real and imaginary parts of its dielectric coefficient. As the TiB content further increases, the anomalous diffusion zone reappears and the frequency decreases to 8 GHz. At this time, the polarization establishment time is extended to 1.3 × 10^−10^ s. This indicates that TiB can already promote a certain degree of ion polarization inside Ti64 at this content. At the high-frequency range of 12–18 GHz, TiB and Ti64 exhibit a relatively broad resonance phenomenon. After further increasing the TiB content, two anomalous dispersion phenomena appeared in the B4 sample, at 10.5 GHz and 15.2 GHz, respectively, corresponding to a polarization time of 9.5 × 10^−11^s and 6.6 × 10^−11^s. It can be concluded that there are two polarization forms in the B4 sample, similar to ion polarization in the Ti64 lattice at 10.5 GHz as in the B3 sample, and electron polarization induced by TiB at 15.2 GHz. Based on the above data, it can be analyzed that the addition of TiB is different from B_4_C. Due to the existence of only electronic polarization, when the two interact with each other, the electrons in TiB at the interface can increase the electronic polarization rate in Ti64, resulting in an increase in its dielectric coefficient. At the same time, as the TiB content further increases, it begins to affect the polar atoms in Ti64. When the frequency of ion polarization of the polar atoms does not match the electron polarization frequency of Ti64 itself, the polarization intensity of both weakens. When the TiB content is further increased, the ion polarization phenomenon begins to appear in Ti64. At the highest TiB content, two different polarization forms can appear inside Ti64.

From Figure 5c,d, it can be seen that TiB increases the electron polarization in Ti64 while exhibiting strong magnetic losses at high frequencies of 12–18 GHz. This is because when the electrons inside Ti64 resonate with those in TiB, the shift in the electron cloud can cause electromagnetic conversion inside the lattice. This phenomenon further increases the mixing of TiB and Ti64, and TiB can effectively enhance the electron polarization intensity of Ti64. It also changes the internal electron polarization establishment time of Ti64, causing resonance within this frequency band and enhancing the loss ability of electromagnetic waves.

**Figure 6:**
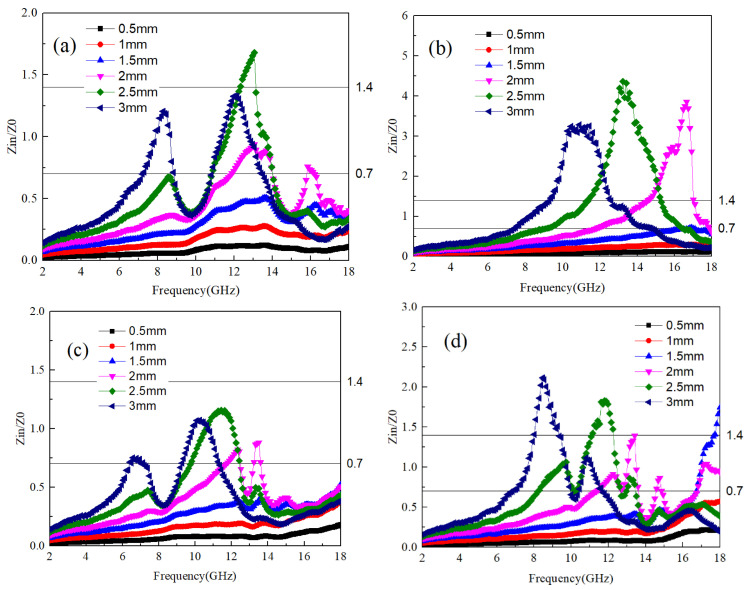
The impedance values of Ti64/TiB with different ratios; (**a**–**d**) correspond to the impedance values of B1~B4 samples at different thicknesses, respectively.

The impedance characteristics of samples B1 to B4, as illustrated in Figure 6a–d, exhibit a correlation with the variation in B_4_C content. With increasing sample thickness, the electromagnetic frequency associated with the impedance value shifts towards lower frequencies. Notably, unlike B_4_C, samples B1, B3, and B4 demonstrate a significantly widened impedance matching frequency band, with each sample exhibiting the best impedance value at a thickness of 2 mm or more.

The optimal impedance matching frequencies for samples B1 and B2 are found to be within the range of 7–9 GHz and 11–14 GHz, respectively, while for sample B3, it falls within 9–12 GHz. The relationship between frequency band and thickness is more pronounced for sample B4, where a thickness of 3 mm corresponds to 7–9 GHz, 2.5 mm to 8–12 GHz, and 2 mm to 11–18 GHz.

Based on these observations, a discernible pattern emerges: within Ti64, an increase in TiB content corresponds to higher electromagnetic wave frequency bands associated with optimal impedance. When TiB content is low, changes in the optimal impedance frequency band with thickness are negligible. However, with increased TiB content, particularly at TiB:Ti64 = 1:10, the impact of sample thickness on the electromagnetic wave frequency band corresponding to the optimal impedance value becomes more pronounced, with thicker samples corresponding to lower electromagnetic wave frequency bands.

**Figure 7:**
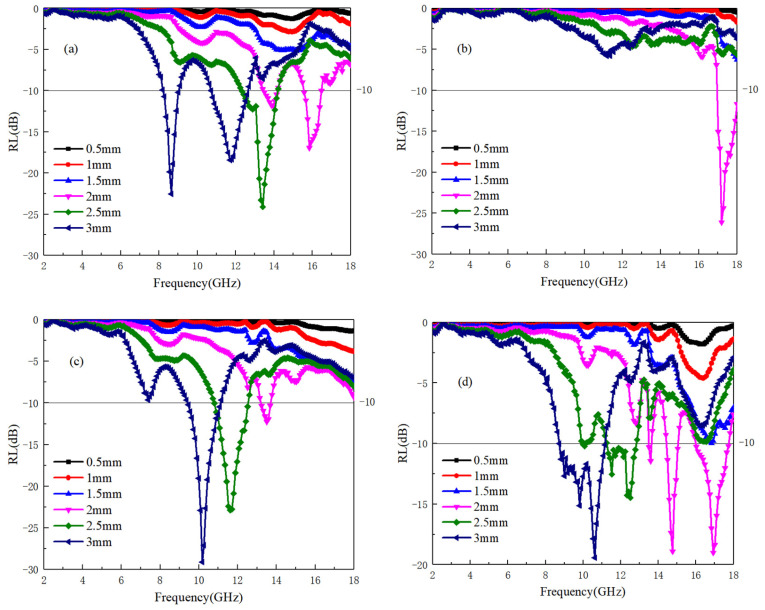
The impedance values of Ti64/TiB at different ratios; (**a**–**d**) correspond to the reflectivity of B1~B4 samples at different thicknesses, respectively.

Figure 7a–d illustrates the electromagnetic wave reflectivity of TiB when mixed with Ti64. As shown in Figure 7b, the B2 sample exhibits poor electromagnetic wave reflectivity due to its low levels of dielectric and magnetic losses. Conversely, for the 2 mm sample, a higher absorption value is observed at 17–18 GHz, attributed to the occurrence of electromagnetic conversion across all samples within this frequency band. Additionally, the B2 sample demonstrates a favorable impedance matching frequency band at 17–18 GHz, contributing to its effective absorption performance within this frequency range.

For the B1, B3, and B4 samples, it can be observed that when the TiB content is low (B1 −3 mm), the optimal absorption frequency band is divided into two parts: 8–9 GHz and 11–13 GHz. However, according to the data in Figure 5b, the B1 sample has significant dielectric loss at 7–13 GHz. Combining with the conclusion in Figure 6a of the impedance matching frequency band, it can be concluded that the impedance mismatch caused by the low dielectric real part value of the B1 sample in this frequency band weakens the absorption performance in the modified frequency band. For the B3 sample, although it has a high dielectric real part value and strong dielectric loss at 6–10 GHz, its high dielectric real part value in this frequency band also leads to a low impedance value for electromagnetic waves entering the sample and unable to achieve effective loss. However, once the impedance reaches the matching frequency band, the ability to lose electromagnetic waves immediately increases. Therefore, for the B3 sample, there is the best electromagnetic wave reflectivity in the 9–11 GHz frequency band, which is −30 dB. From the above conclusion, it can be seen that the B4 sample can perfectly avoid defects in B1 and B3 samples. At 8–12 GHz, there is a certain dielectric loss, and the value of the real dielectric part does not decrease significantly. Therefore, it has good impedance matching in this frequency band. For B3 −3 mm samples, the effective absorption frequency band is 8–12 GHz, with a minimum reflectivity of −20 dB, which can meet the electromagnetic absorption ability and has a wide absorption frequency band.

In summary, the addition of TiB to Ti64 can improve its dielectric performance and alter the inherent electronic frequency of Ti64. During polarization, TiB: Ti64 has a higher dielectric loss at 1:20. However, during the loss process, the real part of the dielectric coefficient decreases rapidly, resulting in a narrower absorption bandwidth. When TiB: Ti64 = 1:10, although the dielectric loss ability decreases, the real part of the dielectric decays slowly, enabling impedance matching over a wide frequency range, resulting in a wider effective absorption bandwidth.

### 3.3. Electromagnetic Performance of Ti64/TiC Mixture

**Figure 8:**
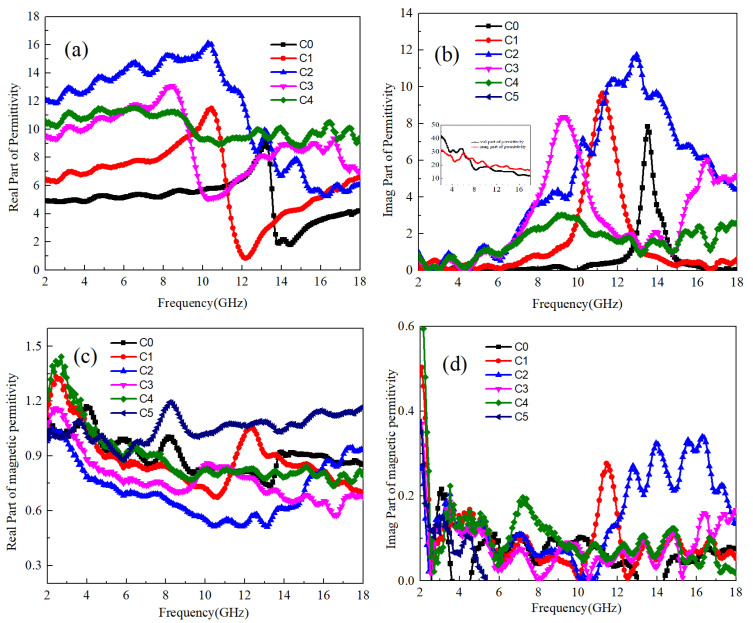
The electromagnetic parameters of Ti64/TiC with different ratios; (**a**) real part of dielectric coefficient, (**b**) imaginary part of dielectric coefficient, (**c**) real part of magnetic permeability, (**d**) imaginary part of magnetic permeability.

The electromagnetic parameters of TiC and Ti64 mixed samples are depicted in Figure 8a–d. As shown in Figure 8b, the TiC samples exhibit high values of both the real and imaginary parts of the dielectric coefficients, indicating strong polarization ability and significant dielectric loss. Furthermore, analysis of the real and imaginary parts of TiC suggests the absence of anomalous dispersion phenomena, implying that the polarization mode of TiC primarily involves electron polarization.

From Figure 8a, it is evident that for the TiC:Ti64 mixed mass ratio of 1:100 (C1 sample), the dielectric coefficient of the sample gradually increases between 2–11 GHz, peaking at 11 around 10 GHz. Subsequently, as the electromagnetic field frequency rises, the C1 sample experiences a significant decrease between 11–12 GHz, followed by a gradual increase between 12–18 GHz. Notably, the presence of an anomalous dispersion phenomenon is observed at 11.5 GHz, with a polarization relaxation time of 8.7 × 10^−11^ s. In comparison to Ti64 samples, the strong electronic polarization ability of TiC leads to an improvement in the electronic polarization rate within the Ti64 lattice. This enhancement not only increases the electronic polarization of Ti64 but also prolongs the time required for establishing electronic polarization. Furthermore, with further increases in TiC content, the dielectric coefficient of the C2 sample exhibits a significant increase. Specifically, within the 2–12 GHz range, it reaches its highest real part at around 17. As the electromagnetic field frequency continues to increase, the dielectric real part of the C2 sample begins to decrease at 11–18 GHz, stabilizing at around 6 near 18 GHz. Moreover, compared to the dielectric imaginary part of the C2 sample, peaks are observed at 11.5 GHz and 12 GHz, accompanied by a significant decrease in the dielectric real part within the corresponding frequency band. This indicates the occurrence of anomalous dispersion phenomena at both frequencies, suggesting resonance phenomena during electron polarization for TiB and Ti64 at this ratio.

The mutual influence between the two can be verified by the simultaneous decrease in the real and imaginary parts of the dielectric in the frequency range of 14~18 GHz. As the TiC content further increases, the dielectric coefficient of the C3 sample decreases compared to the C2 sample in the frequency range of 2–9 GHz, with a dielectric coefficient close to about 10. This indicates that at this ratio, there is a mismatch between the electron polarization in TiB and the electron polarization establishment time in Ti64. Combined with the imaginary part of the dielectric of the C3 sample, it can be seen that abnormal dispersion occurs near 9.0 GHz, with a polarization establishment time of 1.1 × 10^−10^ s. Similar to TiB, when the content of TiC doping is higher than the electron resonance of both, TiC begins to affect the atomic vibration inside Ti64, promoting the transition from electron polarization to ion polarization inside Ti64, resulting in a decrease in its polarization establishment time. As the TiC content further increases, there is no significant change in the dielectric coefficient of C4 samples in the 2–8 GHz frequency band. However, at 9 GHz frequency, its anomalous dispersion phenomenon becomes less obvious. Based on the conclusion of TiB, at this content, the frequency of ion polarization induced by TiC inside Ti64 does not match the electronic polarization frequency of Ti64 itself. Therefore, the establishment time of polarization becomes less obvious due to the mutual influence of the two. For TiC, due to its strong electronic polarization ability, when its content is high, although there is the influence of internal electron and ion polarization of Ti64, it has little effect on its electronic polarization rate. Therefore, its dielectric real part is still high.

In terms of magnetic permeability, as illustrated in Figure 8c,d, the real part of samples C1 to C4 remains approximately 1. However, a notable observation is made in the imaginary part, where the C1 sample exhibits magnetic loss at 10–12 GHz, while the C2 sample similarly demonstrates magnetic loss within the range of 11–18 GHz. Based on the foregoing analysis, it is evident that the enhanced electron polarization ability observed in the C1 and C2 samples within this frequency band leads to the occurrence of resonance phenomena, consequently resulting in electromagnetic conversion.

**Figure 9:**
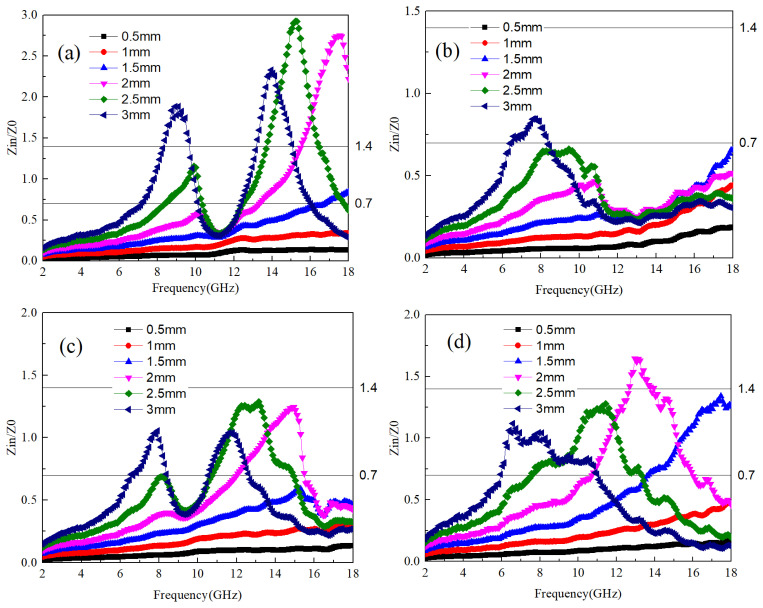
Shows the impedance values of Ti64/TiC with different ratios, (**a**–**d**) correspond to the impedance values of B1~B4 samples at different thicknesses, respectively.

Figure 9a–d illustrate the impedance characteristics of the samples. For the C1 sample, electromagnetic conversion occurs within the 10–12 GHz frequency band, leading to a significant decrease in the real part of the dielectric within that range. Consequently, the impedance value in this frequency band is lower than the matching value, similar to the observations made in B_4_C and TiB samples. Moreover, as the thickness increases, the impedance matching frequency band of the C1 sample shifts towards lower frequencies. Particularly for the 3 mm thickness C1 sample, the impedance matching frequency band appears relatively broad.

Conversely, for the C2 sample, characterized by large values of both the real and imaginary parts of the dielectric, the magnetic permeability is low, resulting in impedance mismatch. Consequently, the impedance is only within the 6–9 GHz frequency band for a thickness of 3 mm. However, the C3 and C4 samples exhibit a broad impedance matching frequency band within the thickness range of 2–3 mm, particularly noticeable in the C4 sample. Specifically, at 1.5 mm thickness, the impedance matching frequency band spans 14–18 GHz, while at 2 mm, it extends from 11–16 GHz. Moreover, at thicknesses of 2.5 mm and 3 mm, the impedance matching frequency band ranges from 8–13 GHz and 6–11 GHz, respectively. Based on the analysis of impedance matching, it can be deduced that although the C4 sample exhibits low dielectric loss, its broad impedance matching frequency band makes it well-suited for specific absorption applications.

**Figure 10:**
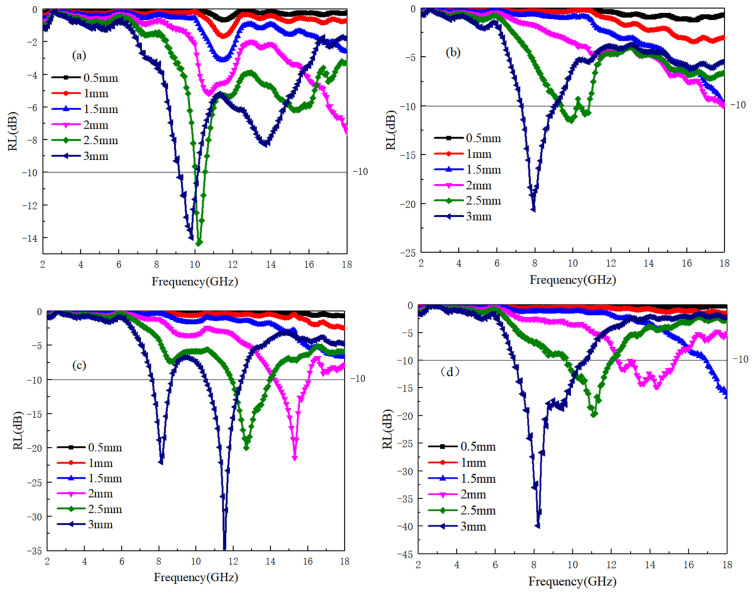
The impedance values of Ti64/TiC with different ratios; (**a**–**d**) correspond to the reflectivity of C1~C4 samples at different thicknesses, respectively.

Figure 10a–d present the reflectance results of samples C1 to C4. While the dielectric loss frequency band of the C1 sample is relatively broad, an analysis of the impedance matching curve reveals that only the 9–11 GHz frequency band satisfies both dielectric loss and impedance matching criteria. Consequently, as depicted in Figure 10a, only the reflectance within this frequency band is below −10 dB, meeting the requirements for effective absorption.

As the TiC content increases, the C2 sample exhibits strong polarization and loss capabilities. However, due to impedance matching limitations at only 3 mm thickness, it demonstrates robust electromagnetic absorption ability within the 7–9 GHz range.

In contrast, the C3 and C4 samples, with TiC and Ti64 exhibiting impedance matching across a wide frequency range, demonstrate ideal absorption performance. Specifically, the C3 samples exhibit absorption performance within the thickness range of 2–3 mm and the frequency range of 8–16 GHz. Notably, the 3 mm sample displays the lowest reflection coefficient of −35 dB within the 11.5 GHz frequency range, meeting the frequency requirements of 7–12 GHz below −10 dB. Similarly, for the C4 sample, the effective absorption frequency range is delineated as follows: 16–18 GHz for 1.5 mm thickness, 12–16 GHz for 2 mm thickness, 10–12 GHz for 2.5 mm thickness, and 7–11 GHz (near 8 GHz) for 3 mm thickness, achieving the lowest reflectivity of −40 dB.

## 4. Conclusions

This study has provided comprehensive insights into the electrical properties of Ti64/B_4_C composite materials fabricated via laser direct manufacturing (LDM) by varying the content of B_4_C. Through rigorous experimentation and analysis, several key findings have been established.

- As the content of B_4_C, TiB, and TiC mixed with Ti64 increases, the internal polarization mechanism of Ti64 transitions from electronic polarization to ion polarization. TiC exhibits the strongest electronic polarization ability, enhancing the electronic polarization of Ti64 significantly. Conversely, TiB demonstrates weaker electronic polarization compared to TiC, and when its content surpasses a certain threshold, polarization establishment mismatches occur, leading to a decrease in the dielectric coefficient of Ti64. B_4_C exhibits weak electronic and ion polarization, resulting in a minor improvement in the polarization ability of Ti64, thereby maintaining a low dielectric coefficient.
- Impedance matching analysis reveals that both high and low dielectric real and imaginary parts fail to meet the requirements for optimal impedance matching within Ti64 when B_4_C, TiB, and TiC are mixed with the alloy. However, when the ratio of TiB and TiC with Ti64 reaches 1:20, and the sample thickness ranges between 2.5–3.0 mm, certain impedance matching frequency bands in the range of 7–16 GHz can be achieved. Similarly, when the ratio of B_4_C with Ti64 reaches 1:30, a comparable impedance matching frequency band is observed within the same frequency range.
- Among the three substances, optimal reflectivity is observed when the TiC: Ti64 content exceeds 1:20, with a sample thickness of 2.5–3.0 mm, showcasing strong reflectivity within the frequency band of 7–12 GHz, with a minimum value of −40 dB. Additionally, optimal reflectivity is achieved for TiB samples at a 1:20 ratio, with a minimum reflectivity of −35 dB at a sample thickness of 3.0 mm and a frequency of 10.2 GHz. Conversely, B_4_C exhibits an optimal reflectivity of −27 dB within the frequency range of 8.0–11.5 GHz, when the mass ratio with Ti64 is 1:30 and the sample thickness is 3 mm. However, for a 1:20 content ratio, although the lowest reflectivity drops to −35 dB, the effective bandwidth is relatively narrow.
- The thickness of the sample influences the optimal impedance and reflectivity frequency bands, with both shifting towards the low-frequency direction as thickness increases.

These findings collectively contribute to the understanding of the electrical behavior of Ti64/B_4_C composite materials and provide valuable insights for their practical applications in various fields. Further research endeavors may focus on optimizing the composite formulations and fabrication techniques to harness their full advantage of their potential for customizing structurally functional integrated components in specific applications.

## Figures and Tables

**Table 1:** Ti64/B_4_C sample mass ratio and sample thickness.

	Ti64: B_4_C	M (Ti64)/g	M (B_4_C)/g	Paraffin/g	Thickness/mm
A0		1		0.428	2.42
A1	100:1	0.99	0.001	0.428	3.33
A2	30:1	0.96	0.032	0.428	3.43
A3	20:1	0.95	0.04	0.428	2.57
A4	10:1	0.91	0.09	0.428	3.39
A5			1	0.428	3.84

**Table 2:** Ti64/TiB sample mass ratio and sample thickness.

	Ti64: TiB	M (Ti64)/g	M (TiB)/g	Paraffin/g	Thickness/mm
B0		1		0.428	2.42
B1	100:1	0.99	0.001	0.428	4.16
B2	30:1	0.96	0.032	0.428	3.23
B3	20:1	0.95	0.04	0.428	4.06
B4	10:1	0.91	0.09	0.428	3.77
B5			1	0.428	3.56

**Table 3:** Ti64/TiC sample mass ratio and sample thickness.

	Ti64: TiC	M (Ti64)/g	M (TiC)/g	Paraffin/g	Thickness/mm
C0		1		0.428	2.42
C1	100:1	0.99	0.001	0.428	3.26
C2	30:1	0.96	0.032	0.428	3.77
C3	20:1	0.95	0.04	0.428	3.49
C4	10:1	0.91	0.09	0.428	2.49
C5			1	0.428	3.45

## Data Availability

The original contributions presented in the study are included in the article, and further inquiries can be directed to the corresponding authors.
